# A Large Insertion in bHLH Transcription Factor *BrTT8* Resulting in Yellow Seed Coat in *Brassica rapa*

**DOI:** 10.1371/journal.pone.0044145

**Published:** 2012-09-11

**Authors:** Xia Li, Li Chen, Meiyan Hong, Yan Zhang, Feng Zu, Jing Wen, Bin Yi, Chaozhi Ma, Jinxiong Shen, Jinxing Tu, Tingdong Fu

**Affiliations:** 1 National Key Laboratory of Crop Genetic Improvement, National Center of Rapeseed Improvement in Wuhan, Huazhong Agricultural University, Wuhan, P.R. China; 2 Guizhou Rapeseed Institute, Guizhou Academy of Agricultural Sciences, Guiyang, P.R. China; 3 Vegetable Research Institute, Guangdong Academy of Agriculture Sciences, Guangdong, P.R. China; 4 Industrial Crop Research Institute, Yunnan Academy of Agricultural Science, Kunming, P.R. China; University of Michigan, United States of America

## Abstract

Yellow seed is a desirable quality trait of the *Brassica* oilseed species. Previously, several seed coat color genes have been mapped in the *Brassica* species, but the molecular mechanism is still unknown. In the present investigation, map-based cloning method was used to identify a seed coat color gene, located on A9 in *B. rapa*. Blast analysis with the *Arabidopsis* genome showed that there were 22 *Arabidopsis* genes in this region including *at4g09820* to *at4g10620*. Functional complementation test exhibited a phenotype reversion in the *Arabidopsis thaliana tt8-1* mutant and yellow-seeded plant. These results suggested that the candidate gene was a homolog of *TRANSPARENT TESTA8 (TT8)* locus. *BrTT8* regulated the accumulation of proanthocyanidins (PAs) in the seed coat. Sequence analysis of two alleles revealed a large insertion of a new class of transposable elements, *Helitron* in *yellow sarson*. In addition, no mRNA expression of *BrTT8* was detected in the yellow-seeded line. It indicated that the natural transposon might have caused the loss in function of *BrTT8*. *BrTT8* encodes a basic/helix-loop-helix (bHLH) protein that shares a high degree of similarity with other bHLH proteins in the *Brassica*. Further expression analysis also revealed that *BrTT8* was involved in controlling the late biosynthetic genes (LBGs) of the flavonoid pathway. Our present findings provided with further studies could assist in understanding the molecular mechanism involved in seed coat color formation in *Brassica* species, which is an important oil yielding quality trait.

## Introduction

*B. rapa* (AA) is an important crop and a model plant for studying *Brassica* genome evolution. Yellow seed is a desirable quality trait of the *Brassica* oilseed species. Compared with their dark-seeded counterparts, yellow seeds of *Brassica* have inherent advantages, such as higher oil content [1]–[3]. *Yellow sarson* (*Brassica rapa var.trilocularis*) is a valuable yellow-seeded variety of *B. rapa* in India. Moreover, *Yellow sarson* is a major yellow-seeded germplasm; used to create several artificial synthetic yellow-seeded varieties of *B. napus* [4]–[5]. Presently, it has been documented that there are two genes involved in controlling the seed color in *B. rapa* [6]–[8]. Seed color genes have been mapped and cloned in *B. rapa* during the past decade [9]–[11], but the molecular mechanism of the seed color formation has not been elaborated in *B. rapa*.

Map-based cloning has been performed on several genes in the *Brassica* species [12]–[13], but few reports describe the cloning of seed color genes. Additionally, the physiological functions of seed color gene in the *Brassica* species are still largely unknown. However, many seed color genes from other plant species such as maize, *Arabidopsis* and rice have been successfully cloned for genetic and molecular analyses. These seed color genes mainly correspond to enzymes and regulatory factors that participate in the flavonoid biosynthesis [14]–[18]. The main enzymes that are involved in the flavonoid synthesis pathway include chalcone synthase (CHS), chalcone isomerase (CHI), flavonoid 3-hydroxylase (F3H), flavonoid 3′-hydroxylase (F3′H), dihydroflavonol 4-reductase (DFR), flavonol synthase (FLS), leucoanthocyanidin reductase (LAR), leucocyanidin dioxygenase (LDOX), anthocyanidin reductase (ANR), peroxidase (POD), and polyphenol oxidase (PPO).

To date, it has been established that the transcriptional regulation of the structural genes for flavonoid biosynthesis is controlled by MYB and basic helix–loop–helix (bHLH) transcription factors, together with WD40 proteins. Recently, the bHLH transcription factors are found to be involved in the transcriptional regulation of the flavonoid pathway [19]–[20]. The first bHLH transcription factor regulating the flavonoid pathway is identified in maize which regulates the anthocyanin biosynthesis pathway in seed [21]. The bHLH transcription factors *Rc*/*Rd* specifically control proanthocyanidins (PAs) synthesis in the grain pericarp in rice [22]. bHLH proteins in *Arabidopsis* such as TT8 control both anthocyanin and PA pathways [18], and is required for normal expression of the flavonoid “late” biosynthetic genes (LBGs) including *DIHYDROFLAVONOL 4-REDUCTASE (DFR)* and *BANYULS (BAN)*. DFR is the first enzyme leading to the production of anthocyanidin, and the *BAN* encoded ANR, the core enzyme in PA biosynthesis [23]–[24]. In addition, through the yeast two- and three-hybrid experiments, TT8 (bHLH), TT2 (MYB), and TTG1 (WD40) can form a ternary complex which is involved in the regulation of *BAN* expression, and the expression of *TT8* itself is also controlled by different combinations of MYB and bHLH factors in the seed coat of *Arabidopsis thaliana* [25]–[26]. These data clearly indicate that bHLH transcription factors can regulate one or more branches of the flavonoid pathway, and the structural and functional similarities of transcription factors may slightly differ depending on its species.

Using the ethyl methanesulfonate (EMS) treatment or T-DNA insertion, a series of seed coat color mutants in *Arabidopsis thaliana* have been produced. However, in natural mutations, seed coat color change is due to a variety of factors: for example, changes in the length of the sequences that contain the insertion/deletion mutations in flavonoid biosynthetic genes, such as the *Rc* in rice and *TTG1* homolog in *B. rapa* [9], [16]. Another factor is endogenous RNA interference (RNAi), which occurs in the *CHS* genes in soybean [27]. In addition, it is well known that the transposable elements affect the seed coat color in maize. The transposable elements participated in pigment metabolism have also been reported in other plant species [28]–[29].

In this study, we reported the isolation and functional characterization of the *BrTT8*. *BrTT8* regulated the accumulation of pigment in the seed coat. The sequence analysis of two alleles showed that a transposable element could affect the seed coat color. *BrTT8* encodes a protein exhibiting strong similarity with other bHLH proteins in *Brassica*. Additional experiments also demonstrated that *BrTT8* modulated the expression of flavonoid “LBGs”. These results have provided useful information which could assist in the current understanding of the molecular mechanism of seed coat color formation in yellow-seeded *Brassica* crops.

## Results

### Phenotypic and genetic characterization of seed color in *B. rapa* BC_5_ population

**Figure 1:**
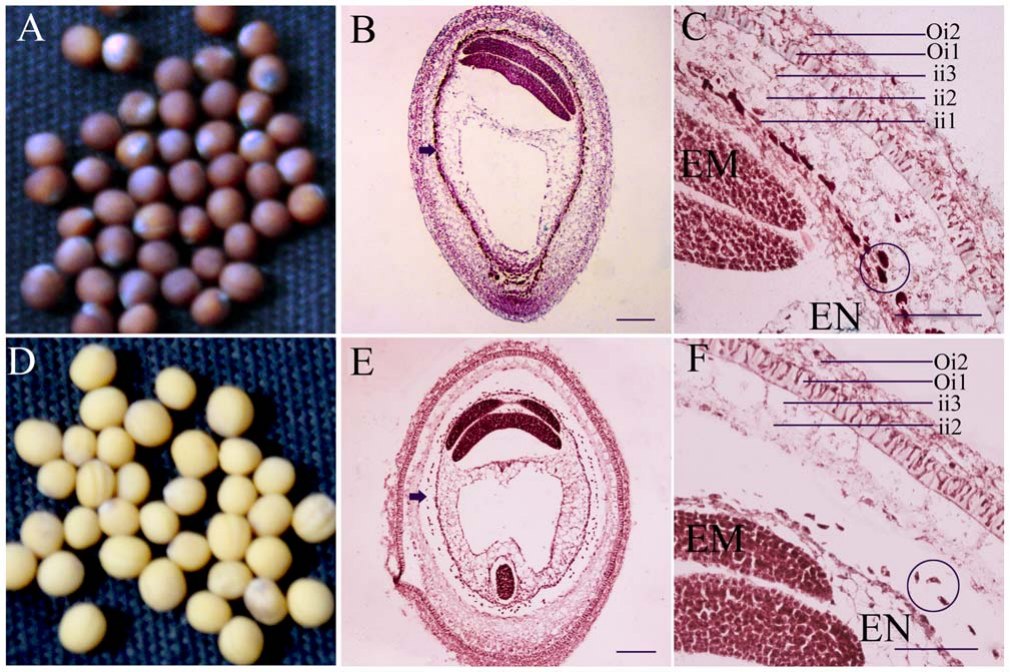
Seed coat structure of the *B. rapa*. (A) The black seeds of the *B. rapa*. (B) The whole black seed treatment with Safranine O and Fast Green. Arrowheads show the Phenolic compounds are localised in the seed coat of immature seed (20 days after flower). (C) Magnified image of (B). Phenolic compounds stain red, formed granules are localised in the endothelium cell of the ii1 layer (circle in C). (D) The yellow seeds of the *B. rapa*. (E) The whole yellow seed treatment with Safranine O and Fast Green (20 days after flower). Phenolic compounds are absent in the endothelium of the yellow seed (arrowhead in E). (F) Magnified image of (E). There are only unknown fragmented structures in the region of the endothelium cell layer (circle in F). EM, embryo; EN, endosperm; ii, inner integument; Oi, outer integument. Bar(N) = 100 µm for (B) and (E); 50 µm for (C) and (F).

The population BC_5_ was developed by backcrossing 3H219 (black-seeded parent) as a donor to the *Yellow sarson* (yellow-seeded parent). The black-seeded plant exhibited brown color seeds (Figure 1A), but the yellow-seeded individuals showed bright yellow color seeds (Figure 1D). Histological analysis of the immature seeds showed that there were three inner integument cell layers (ii1, ii2, ii3) in the black seed coat (Figure 1C), but the PA-accumulating cell layer (iil) was completely absent in yellow seed coats, and there were some unknown fragments present (Figure 1F) when stained with Safranine O and Fast Green treatment. This shown that the absence of PAs was responsible for yellow color of the seeds.

The genetic analysis showed that the yellow seed trait was recessive, monogenic, and maternal. A total of 1183 individuals were obtained from the BC_4_ black-seeded individuals, of which 565 plants were yellow-seeded and 618 plants were black-seeded. This segregation of yellow and black was consistent with the expected Mendelian ratio of 1∶1 (χ^2^ = 2.28, χ^2^_0.05_ = 3.84), confirming that only one seed color gene is present in the BC_5_ population.

### Identification of candidate gene for seed coat color trait

**Figure 2:**
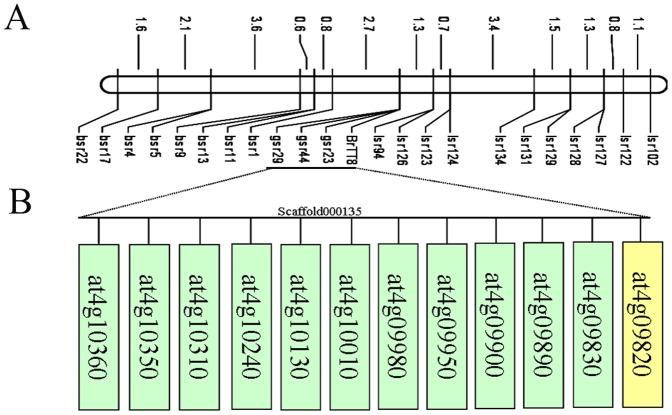
Mapping of the *BrTT8* gene. (A) The genetic linkage map of the *BrTT8* gene. The markers gsr23, gsr44, and gsr29 are derived from Scaffold000135. (B) BLAST analysis with the *Arabidopsis* genome showed that Scaffold000135 shared similarity with a region on chromosome 4. The rectangles containing *Arabidopsis* genes present several representative genes (E<10^−30^) in this region.

AFLP markers were screened in the BC_1_ population, and the markers that tightly linked to the seed color gene were selected for sequencing. Blast analysis suggested that the molecular marker EA11MG10 shared a very high sequence similarity with the BAC KBrB072E02, which is located in the linkage Group A9 (e-171). Therefore, BAC sequences within the target region were collected from the publicly available *B. rapa* genomic sequences (http://www.brassica-rapa.org/BRGP/index.jsp) and BRAD (http://brassicadb.org/brad/). Based on the BAC sequences, SSR primers were designed using the SSR finder tool. Using 22 SSR markers from the A9 BAC sequence of *B. rapa* (Table S1), the seed coat color gene was located on linkage group 9 corresponding with the two molecular markers lsr126 and bsr1 that are located at 1.3 cM and 2.7 cM, respectively (Figure 2A). In addition, we developed co-separation SSR molecular markers that linked to the seed coat color gene from the Scaffold000135 sequence on A9. Blast analysis using the *Arabidopsis* genome showed that the sequence was similar to a region of chromosome 4 (Figure 2B). There are 22 *Arabidopsis* genes in the region including *at4g09820* to *at4g10620*. It has been well established that *at4g09820* (*TT8*) is involved in the regulation of flavonoid biosynthesis in *Arabidopsis*, and *tt8* mutations possess transparent testa [18]. Moreover, the *TT8 Brassica* homolog showed no recombination with the seed coat color gene. These results suggested that the candidate gene was likely a *TT8* homolog.

### Large insertion in the yellow-seeded line

**Figure 3:**
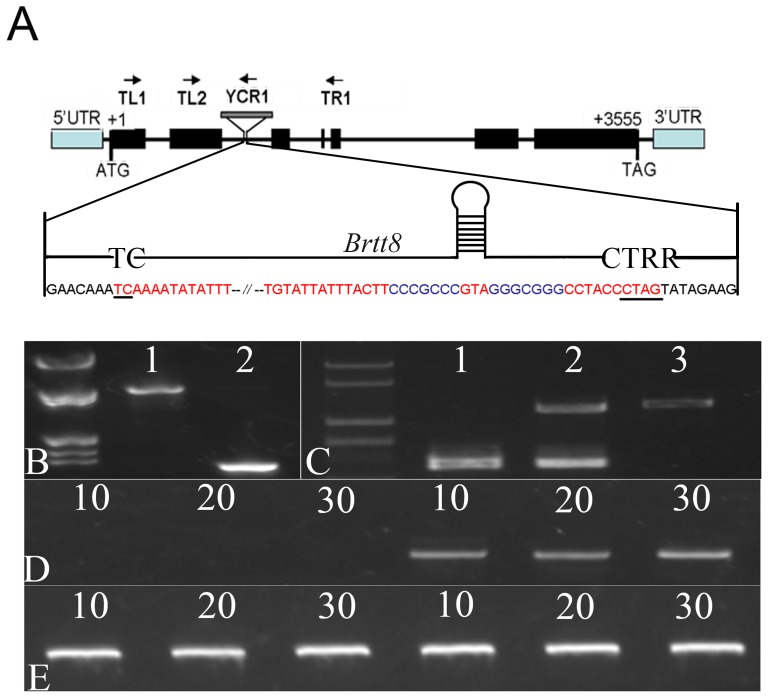
Differences in DNA and mRNA expression. (A) The insertion location is shown in gray rectangles in the ORF of the *BrTT8*. The black rectangles represent the exons, and TL1, TL2, TR1 and YCR1 are the primers that were developed from the corresponding exon sequences and insert sequence. The arrows are used to indicate the directions of the primers. The insertion sequences and flanking *BrTT8* intron 2 are shown in red and black, respectively. The conserved sequences at the termini of the element are underlined. Palindromic sequences that are capable of forming a hairpin are shown in blue. (B) The amplification products of the genomic DNA of the yellow-seeded (1) and black-seeded line (2) using the primers TL1 and TR1. (C) The primers TL2, TR1 and YCR1 amplified the genomic DNA from the three genotypes: (1) the homozygous yellow-seeded, (2) heterozygous black-seeded, (3) homozygous black-seeded plants. (D) mRNA levels in the immature seeds of the yellow-seeded line (three on the left) and black-seeded line (three on the right). The numbers 10, 20, and 30 signify the number of days after pollination. (E) 18S control.

The molecular data allowed us to design specific primers for the cloning of the full-length *TT8* ortholog by PCR amplification. For this purpose, we used the primers Tu-L and Tu-R (Table S2), which were designed from the predicted homologous sequence of *at4g09820* in the Scaffold000135. The amplification of the corresponding genomic sequence from the black-seeded line resulted in a 5420-bp fragment (Figure S1) that spanned the entire 3555-bp (Figure S2) putative *TT8* homolog open reading frame (Figure 3A). However, using the same primers, no amplification was observed in the yellow-seeded line. The primers TL1 and TR1 (Table S2), based on the *B. rapa* sequences showed high homology with the first and fifth exon sequences of *Arabidopsis*, respectively were used to amplify a fragment of that gene in yellow-seeded. Surprisingly, a fragment was amplified that was much larger in length than anticipated (Figure 3B). It was speculated that there was a large insertion in this region.

Due to its complex secondary structure, the insertion sequence was acquired through restriction enzyme digestion and sequencing. The sequence (Figure S3) analysis showed that the inserted fragment contained the structural characteristics of a recently discovered class of transposable elements in eukaryotes, termed *Helitron*. The insertion was 4320 bp, starting with 5′ TC, ending with 3′ CTAG, and containing two short palindromic sequences that were possibly formed by the 17-bp hairpin that was located near its 3′ terminus (Figure 3A).

For further verification, we again designed the primer TL2 in the second exon and the primer YCR1 (Table S2) in the insertion sequence. For the three genotypes, the same fragment was amplified in the homozygous yellow-seeded line and heterozygous black-seeded line (Figure 3C1, 2), but no fragment was amplified in the homozygous black-seeded line (Figure 3C3). We determined the level of mRNA expression in the seeds of the yellow-seeded line in comparison with that of the black-seeded line, to detect the expression changes of the *Brassica TT8* orthologue due to the insertion. There was no mRNA detected in the immature seeds of yellow-seeded line (Figure 3D). This indicated that the inserted fragment disturbed the normal transcription of *BrTT8* in the yellow-seeded line.

### *BrTT8* encodes a bHLH-Domain protein

**Figure 4:**
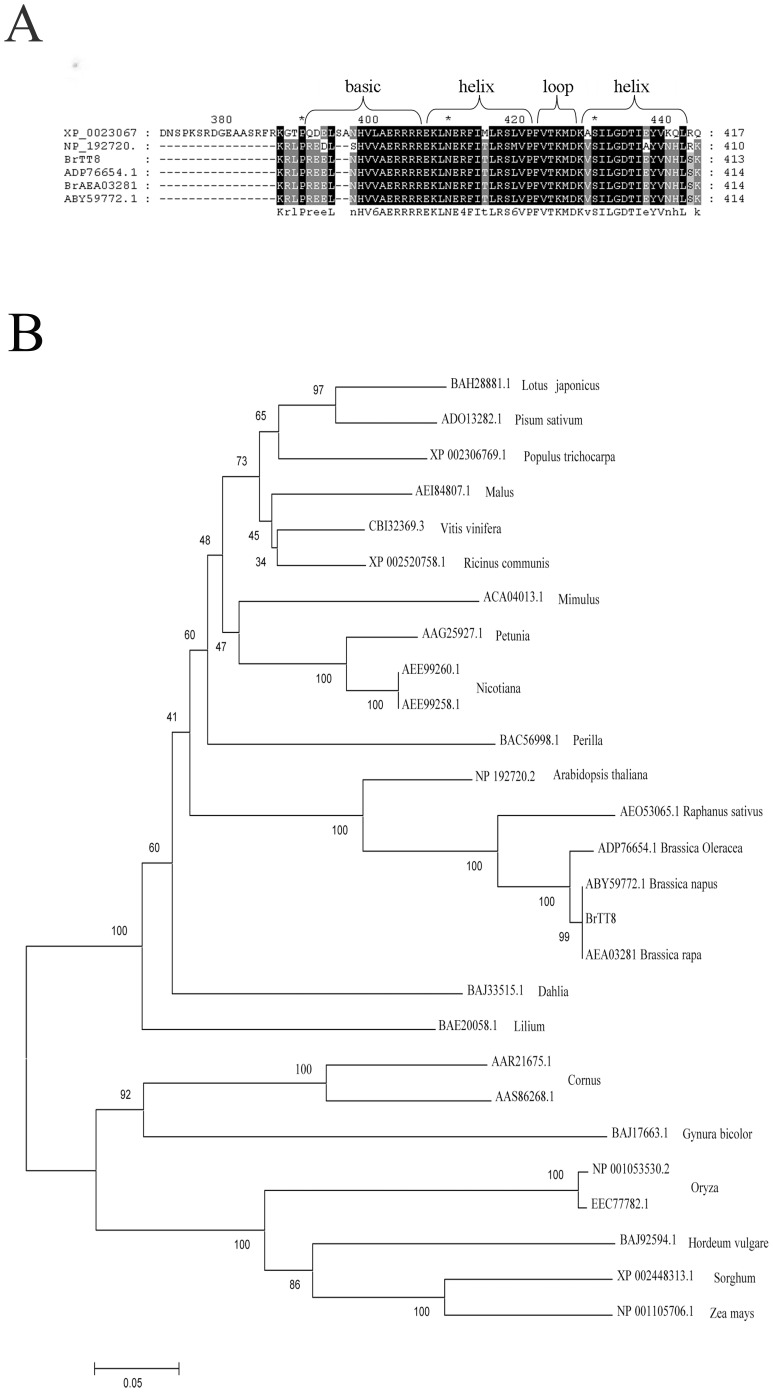
BrTT8 shows features of a bHLH DNA-Binding domain protein. (A) Amino acid comparison of the bHLH domain of *BrTT8*, *B. napus* (ABY59772.1), *B. rapa* (AEA03281), *B. oleracea* (ADP76654.1), *Arabidopsis* (NP_192720.2) and *Populus* (XP_0023067). (B) Dendrogram of the relationships among the bHLH domains from several bHLH-related proteins. For the construction of the tree, the BrTT8 protein sequence and other selected bHLH-related proteins were used. The sequence similarity was calculated using the MEGA programme to generate a branching pattern. The numbers below the branches indicate the percentages of bootstrap support after 1000 replicates. The sequences used are *Brassica rapa* AEA03281, *Arabidopsis* NP_192720.2(AtTT8), *Brassica napus* ABY59772.1, *Brassica oleracea* ADP76654.1, *Populus* XP_002306769.1, *Vitis vinifera* CBI32369.3, *Lotus* BAH28881.1(LjTT8), *Raphanus* AEO53065.1, *Pisum sativum* ADO13282.1, *Perilla* BAC56998.1(F3G1), *Ricinus* XP_002520758.1, *Malus* AEI84807.1, *Petunia* AAG25927.1(AN1), *Nicotiana* AEE99260.1, *Nicotiana* AEE99258.1(NtAN1b), *Hordeum vulgare* BAJ92594.1, *Lilium* BAE20058.1, *Sorghum* XP_002448313.1, *Dahlia* BAJ33515.1, *Oryza* NP_001053530.2, *Zea mays* NP_001105706.1, *Cornus* AAR21675.1, *Cornus* AAS86268.1, *Oryza* EEC77782.1, *Mimulus* ACA04013.1, and *Gynura bicolor* BAJ17663(GbMYC1).

The *BrTT8* gene encodes a putative bHLH protein consisting of 520 amino acids (Figure S4) with a predicted molecular weight of 59.5 kD, and a pI of 5.45 (http://www.expasy.org/tools/protparam/). The BrTT8 protein sequence contains a typical bHLH signature near the C terminus that corresponds with a putative binding domain (http://www.expasy.ch/tools/scanprosite/). The bHLH structure and most of its invariant amino acid residues are conserved, consisting of a basic region (14 amino acids) and two α-helices separated by a loop of variable length (Figure 4A).

Distance analysis of bHLH sequences was performed (Figure 4B), which suggested the existence of 4 groups. *BrTT8* encodes the bHLH protein belonging to group 3, which occurs in five species of Brassicaceae: AEA03281, containing the bHLH protein found in *Brassica rapa*, ABY59772.1, with the bHLH protein from *Brassica napus*, ADP76654.1, with the bHLH protein in *Brassica oleracea*, AEO53065.1, with the bHLH protein from *Raphanus sativus*, and NP 192720.2(AtTT8), containing the TT8 protein that is found in *Arabidopsis thaliana*. In addition, within the large family of plant bHLH-domain proteins, the four protein sequences of the *Brassica* shared the highest similarity with the BrTT8.

### Functional complementation of the mutation

**Figure 5:**
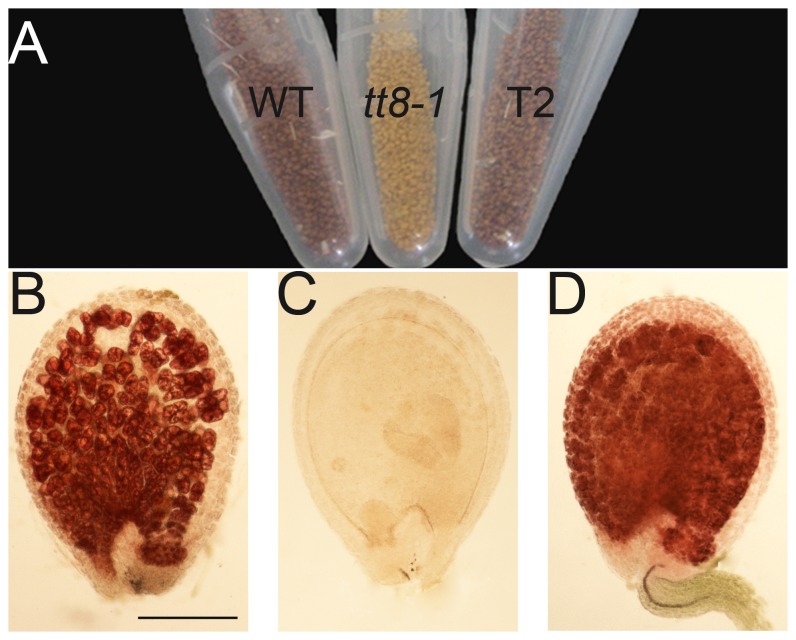
Complementary test and PAs localization in T_2_ seeds. (A) Seeds of the TT8 wild-type genotype (left), mutant (middle) and T_2_ progeny of a tt8 homozygous-transformed *Arabidopsis* with the *BrTT8* genomic region (right). (B–D) Detection of PAs and their precursors in immature seed (heart stage) treated with vanillin HCl. The vanillin test stains the PAs and their precursors (leucoanthocyanidins and catechins) red in the endothelium of the wild type (B); the complete absence of these compounds in *tt8-1* immature seed (C); the recovery of these compounds in T_2_ immature seeds (D). Bar (N) = 100 µm for the (B), (C) and (D).

A modified pC2300 vector was used to conduct a functional complementation experiment to confirm that the phenotype was caused by a mutation in the *Brassica TT8* ortholog gene. 5420-bp genomic clone containing the *BrTT8* gene, along with a 1.2-kb promoter and a 0.6-kb 3′ flanking region from the black- seeded line, was introduced into an *Arabidopsis tt8-1* mutant (SALK_030966). The T_2_ seed progeny originating from 25 independent T_1_ kanamycin-resistant transformants exhibited phenotypic reversions in seed color compared with the wild type (Figure 5A). Furthermore, analysis of PAs deposition during seed development was performed by staining PAs and flavan-3-ol precursors with vanillin in the wild type and T_2_. It showed that PAs accumulated in the seed body of the T_2_ (Figure 5D) similar to the wild type (Figure 5B) indicating that the PAs production defect of *tt8-1* mutant was indeed restored by *BrTT8*. Clone contained the *BrTT8* gene was introduced into the yellow-seed line, and a phenotypic reversion was exhibited in one positive transformant. These data demonstrate that the yellow-seeded coat in *B. rapa* was conferred by the mutant of *BrTT8*.

### *BrTT8* regulated flavonoid gene expression in *B. rapa*

**Figure 6:**
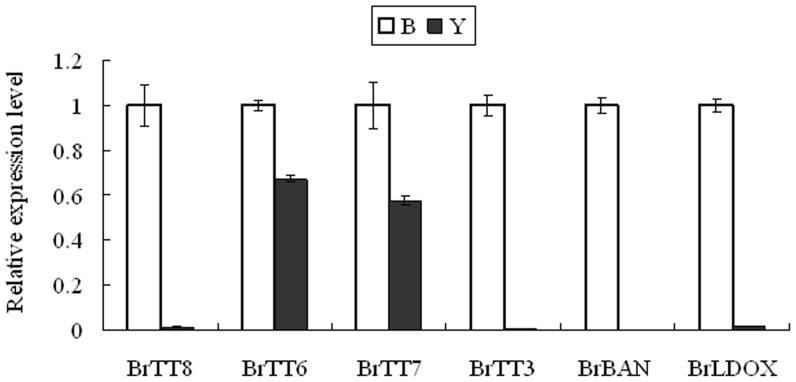
Expression of flavonoid biosynthetic genes in developing seed. Seeds were obtained from the yellow-seeded and black-seeded plants 10 days after pollination. The expression of the different genes was detected by quantitative Real-time PCR. Transcripts for two flavonoid EBGs *BrTT6* (encode F3H, flavanone 3-hydroxylase) and *BrTT7* (encode F3′H, flavanone 3′-hydroxylase), three flavonoid LBGs *BrDFR* (encode dihydroflavonol reductase), *BrBAN* (encode anthocyanidin reductase) and *BrLDOX* (encode LDOX, leucoanthocyanidin dioxygenase) in *B. rapa*. The comparative Ct method was used to calculate the levels of transcripts relative to black-seeded plant. (“B” in the legend is the black seed and “Y” is the yellow seed).

Expression analysis was conducted to compare the effects of *BrTT8* on flavonoid metabolism in *B. rapa* with those of *TT8* in *Arabidopsis thaliana*, both of which modify seed pigmentation pattern. The expression level of five flavonoid genes, from two group EBGs and LBGs, were analyzed during the development of seeds in yellow-seed line and black-seed line, respectively (Figure 6). Quantitative Real-time PCR (QRT-PCR) analysis revealed that the yellow-seeded line contained similar amounts of mRNA as the wild-type for *BrTT6* and *BrTT7* which encoded the flavonoid 3-hydroxylase (F3H) and flavonoid 3′-hydroxylase (F3′H), respectively in the flavonoid metabolism pathway, and have been classified as flavonoid EBGs. Conversely, transcripts of three flavonoid LBGs, *BrDFR*, *BrBAN* and *BrLDOX* (encoded leucocyanidin dioxygenase) were statistically insignificant in the yellow-seeded line. Therefore, our results indicated that *BrTT8* is also involved in the genetic control of flavonoid late metabolism in the developing seed.

## Discussion

### *BrTT8* is essential for formation of normal seed coat color in *B. rapa*

Seed color formation is due to the accumulation of flavonoids, mainly consists of flavonols, anthocyanins, phlobaphenes, isoflavones, and proanthocyanidins [30]. The brown to dark color of the mature seeds were due to the PAs oxidations during seed desiccation that accumulate as colorless compounds in the seed coat [31]–[32]. At present, 20 genes involved in the PA biosynthesis pathway have been identified in the *Arabidopsis thaliana* [30]. However, only a few genes such as *Bna.BAN* and *BnTT2* involved in proanthocyanidin biosynthesis have been identified through genetic and molecular studies in *Brassica* [33]–[34]. *Bna.BAN* genes were located on oilseed rape genetic maps and co-localised with a potential seed color quantitative trait loci. *ProBna.BAN* was activated in proanthocyanidin-accumulating cells, namely the innermost layer of the inner integument [33].

In our study, histological analysis showed that the black color of seeds was also due to the PAs accumulation in the plant endothelium layers of immature seeds (Figure 1B). However, there was no pigment present in the seed coats of the yellow-seeded plants (Figure 1E). Compared with the black-seeded plants, there was no accumulation of red granules in the endothelium layers of yellow seed coat after Safranine O and Fast Green treatments (Figure 1E). In addition, *BrTT8* was necessary for the expression of flavonoid LBGs, such as *LDOX* and *BAN* in the developing seeds (Figure 6), similar to *TT8* in *Arabidopsis* [18], [24]. These results indicated that *BrTT8* was involved in the PA biosynthesis pathway, and the mutation of the seed color in *B. rapa* was due to impairment of the PAs accumulation in the seed coat.

Furthermore, in the yellow seeds of the *B. rapa*, because of the insertion mutation of *BrTT8*, histological analysis showed that the ii1 layer was completely absent (Figure 1F). To our present knowledge, the mechanism of the structural change was still unknown in *B. rapa*. Recently, research has shown that epidermal cell fate, seed-coat development and flavonoid biosynthesis are linked in *Arabidopsis* [35]. Moreover, the bHLH transcription factors have multiple functions in different biosynthetic pathway in plant species [36], so we speculated that the change of cell layer structure in yellow seed coat might be due to *BrTT8* and it also influence the development of PAs accumulation cell layer in the seed coat.

### A natural insertion leads to the mutation of *BrTT8* in *B. rapa*

*Yellow sarson* is not only a valuable yellow-seeded variety of *B. rapa* but also a major yellow-seeded germplasm, used to produce several artificial yellow-seeded varieties in *B. napus* [4]–[5]. Several seed coat color genes have been mapped, but few have been cloned by map-based cloning. Using BC_5_ population, a seed coat color gene was isolated and found to be located in Scaffold000135 on A9 of *B. rapa* which contains a homologous of *TT8* gene (*at4g09820*). *TT8* gene (*at4g09820*) is involved in the regulation of flavonoid biosynthesis in *A. thaliana*.

Interestingly, the sequence analysis of alleles revealed that the seed coat color mutation in *B. rapa*, similar to the *tt8-3* mutation in *A. thaliana*, was caused through insertion in the second intron (Figure 3B and 3C). In *A. thaliana*, no *TT8* mRNA expression was detected in siliques of *tt8-3* [18]. Accordingly, we investigated the expression of *BrTT8* in *B. rapa* and found that no mRNA expression was detected in the immature seeds of the yellow-seeded line (Figure 3D). These data demonstrated that the disturbance in the transcription was caused by the insertion of the second intron.

The sequence analysis showed that the inserted fragment was a recently discovered class of transposable elements in eukaryotes, termed *Helitron*. *Helitrons* are quite large, ranging from approximately 5 to 15 kb. They lack terminal repeats, do not duplicate host insertion sites, and insert consistently between the nucleotides A and T. The *Helitron* structure consists of 5′ TC and 3′ CTRR termini, and a 16- to 20-bp predicted small hairpin structure located 10 to 12 bp upstream of the 3′ end [37]. The insertion was 4320 bp in our study, starting with 5′ TC, ending with 3′ CTAG, and containing two short palindromic sequences that were possibly formed by the 17-bp hairpin located near 3′ terminus (Figure 3A). This shown that the new transposable element is, in all probability, responsible for the changes in seed coat colour in *B. rapa*.

Although, there is no report of *Helitron* transposon in *Brassica*, and *Helitrons* have been found in seven additional flowering plant species [37]. Moreover, *Helitron* transposable elements participated in pigment metabolism has been reported in morning glory [29]. The extensive research on its presence has been done in maize. Researchers have found that the *Helitron* transposon often results in exon shuffling or the duplication of gene sequences [38]–[39]. Moreover, *Helitrons* also participate in the rearrangement and duplication of genomic regions, contributing to the evolution of novel eukaryotic genomic functions [40]–[41].

### *BrTT8* is a highly conserved gene

*BrTT8* in *B. rapa* displayed the typical features of a transcription factor with a bHLH signature at its C terminus. The bHLH domain consists of 50–60 amino acids that form two distinct segments: the basic region which contains 10–15 predominantly basic amino acids, and the helix-loop-helix region which form two predicted amphipathic α-helices that are separated by a loop of variable length [42]. The retrieved amino acid sequences were aligned with those of five other plant speceis in the conserved bHLH region and found that the key amino acid residues were highly conserved in the bHLH domain (Figure 4A). Moreover, the sequences of BrTT8 and other bHLH proteins in *Brassica* species were almost completely identical.

The coding sequence of *BrTT8* from the black-seeded line of *B. rapa* was used to complement the *Arabidopsis tt8* mutant. *BrTT8* completely restored the wild-type phenotype of the *tt8* mutant, suggesting that *BrTT8* has a conserved function (Figure 5A). In addition, BLAST analysis showed that *BrTT8* is a single-copy gene in the *B. rapa* genome. Thus, the analytical results demonstrated that *BrTT8* was highly conserved gene in *B. rapa*.

## Materials and Methods

### Plant materials

Two backcross populations, BC_1_ and BC_5_, were developed by backcrossing *3H219* (black-seeded parent) as a donor to *Yellow sarson* (yellow-seeded parent). Markers linked with the target gene were used to select black-seeded individuals in each generation of backcrossing. The BC_1_ population, containing 202 individuals, was used to identify the amplified fragment length polymorphisms (AFLP) and simple sequence repeats (SSR) markers that are linked to the seed coat color gene. The BC_5_ population, containing a total of 1183 individuals, was used for molecular mapping of the seed coat color gene.

### Markers development

DNA was extracted using a CTAB modified method according to Lei *et al.* (2007) [43]. AFLP analysis was conducted as described by Liu *et al.* (2005) with minor modifications [44]. The two bulks along with the two parental lines were subjected to AFLP analysis. The AFLP fragments cloning and sequence analyses were performed as described by Lei *et al* (2007) [43]. The AFLP sequences were analyzed using BLAST of the National Center for Biotechnology Information (http://www.ncbi.nlm.nih.gov/). Bacterial artificial chromosome (BAC) sequences within the target region were collected from *B. rapa* genomic sequences (http://www.brassica-rapa.org/BRGP/index.jsp) and the *Brassica* database (BRAD) (http://brassicadb.org/brad/). All of the SSR primers were designed using the web-based SSR finder tool (http://www.geboc.org/index). A local linkage map of the region on the chromosome surrounding the gene was drawn using Mapdraw V2.5 [45].

### Comparison analysis and candidate gene cloning

The sequences that contained the co-segregate SSR markers were aligned with the *A. thaliana* genome sequences from the TAIR database (http://www.arabidopsis.org/). The sequences with a threshold value of E<10^−30^ were regarded as homologous loci of the *A. thaliana* genome, and located on a physical map. According to the conserved co-linearity with *A. thaliana*, we developed other co-segregate markers, and finally identified the candidate gene. Based on the BAC sequence that contained the seed coat color gene, we designed the primers and cloned the gene.

### Expression analysis

Black and yellow developing seeds were harvested from 5 plants each for studying the expression analysis. We tagged individual flowers on the primary inflorescence on the first day of flower pollination and again every 10 days. Seeds were removed from the siliques at each stage, including 10_DAP, 20_DAP, and 30_DAP (DAP: days after pollination). The tissue samples were stored in liquid nitrogen, and total RNA was extracted with the highly efficient hot CTAB-LiCl synthesis [46]. 0.4 g of grated seeds was mixed with 4 ml 65°C CTAB in a 10 ml centrifuge tube, and incubated in a 65°C water bath heat-treatment for 30 min. The supernatant was then collected by centrifugation after mixing with an equal volume of chloroform/isoamylalcohol (24∶1) at 4°C. After the addition of 1/4 volume 4 M LiCl, the samples were incubated at −20°C for 2 hours. They were eluted with 1.35 mL diethyl pyrocarbonate-treated water, and then 150 µl NaAc (pH 5.2, 3 M) and 3.75 ml 70% ethanol (freezing-treated) were added. Finally, the RNA was dried and dissolved in the DEPC water. The RNA extracts were converted to first-strand cDNA by using MMLV reverse transcriptase (MBI Fermentas, USA) according to the manufacturer's instruction and the products were diluted 100-fold with sterilized ddH_2_O for subsequent PCR reactions. The RT-PCR was performed in 20_µl reactions using 2_µl of diluted first-strand cDNA as the template. The PCR was conducted using the following settings: 35 cycles with 94°C for 30 sec (3 min for the first cycle), 94°C for 30 s, 58°C for 30 s, and 72°C for 40 s, followed by an extension at 72°C for 5 min, after which samples were held at 4°C. The primers for the RT and Q-PCR tests were in the Table S3.

### GenBank accession numbers

The GenBank accession numbers were as follows (Figure 4): *Brassica rapa* (AEA03281), *Arabidopsis* (NP_192720.2), *Brassica napus* (ABY59772.1), *Brassica oleracea* (ADP76654.1), *Populus* (XP_002306769.1), *Vitis vinifera* (CBI32369.3), Lotus (BAH28881.1), *Raphanus* (AEO53065.1), *Pisum sativum* (ADO13282.1), *Perilla* (BAC56998.1), *Ricinus* (XP_002520758.1), *Malus* (AEI84807.1), *Petunia* (AAG25927.1), *Nicotiana* (AEE99260.1), *Nicotiana* (AEE99258.1), *Hordeum vulgare* (BAJ92594.1), *Lilium* (BAE20058.1), *Sorghum* (XP_002448313.1), *Dahlia* (BAJ33515.1), *Oryza* (NP_001053530.2), *Zea mays* (NP_001105706.1), *Cornus* (AAR21675.1), *Cornus* (AAS86268.1), *Oryza* (EEC77782.1), *Mimulus* (ACA04013.1), and *Gynura bicolor* (BAJ17663).

### Construct preparation

A pCAMBIA2300 vector [47] was digested with the EcoRI and HindIII restriction enzymes (NEB, USA). The seed coat color gene was amplified from the black-seeded lines using high fidelity polymerase (NEB, USA) with the primers Tu-L and Tu-R, and suitable restriction enzyme cleavage sites were added for use with the pC2300 vector. The PCR products from the coding sequences were treated with T4_ligase and mixed with the digested vector. Chemical transformation was used to introduce the mixture of PCR fragments and vector DNA into chemically competent *E. coli* DH5a (Invitrogen, USA). Positive clones were selected through PCR and the insert was confirmed by sequencing.

### Microscopy

Immature seed samples were harvested after 20 days of flowering. Seeds were directly fixed in FAA (Formalin 10 ml, Acetic acid 3 ml, 50% Ethanol 87 ml) for 24 hours. After fixation, the material was dehydrated through a series of graded ethanol solutions (50, 70, 90, 100%). Then the material transferred to the graded chloroform-ethanol solutions (25, 50, 75, and 100%) for transparent processing. Finally, the material was paraffin-embedded after infiltration of graded paraffin solutions at 42°C, 56°C, 60°C each one hour later. The embedded samples were sectioned to a thickness of 8 µm using an automatic microtome (Microm HM 360, Thermo). Selected sections were stained for fast green and counterstain with safranine. The vanillin test [48] was performed by direct incubation of immature siliques samples in a freshly prepared solution of 1% (w/w) vanillin (methanol) in 6 N HCl for 30 min at room temperature.

## Supporting Information

Figure S1 **Genomic sequence the** ***BrTT8*** **.**
(TIF)

Figure S2 **ORF sequence of** ***BrTT8*** **.**
(TIF)

Figure S3 **DNA sequence of the insertion.**
(TIF)

Figure S4 **The amino acid sequence of BrTT8.**
(TIF)

Table S1 **The SSR markers were developed in the study.**
(DOC)

Table S2 **The primers were used in the study.**
(DOC)

Table S3 **The primers were used for Q-PCR.**
(DOC)
