# Supplementary figures and images for: A Large Insertion in bHLH Transcription Factor BrTT8 Resulting in Yellow Seed Coat in Brassica rapa
Source: PLoS One. 2012 Sep 11;7(9):e44145. doi: 10.1371/journal.pone.0044145 (PMC3439492; doi:10.1371/journal.pone.0044145)

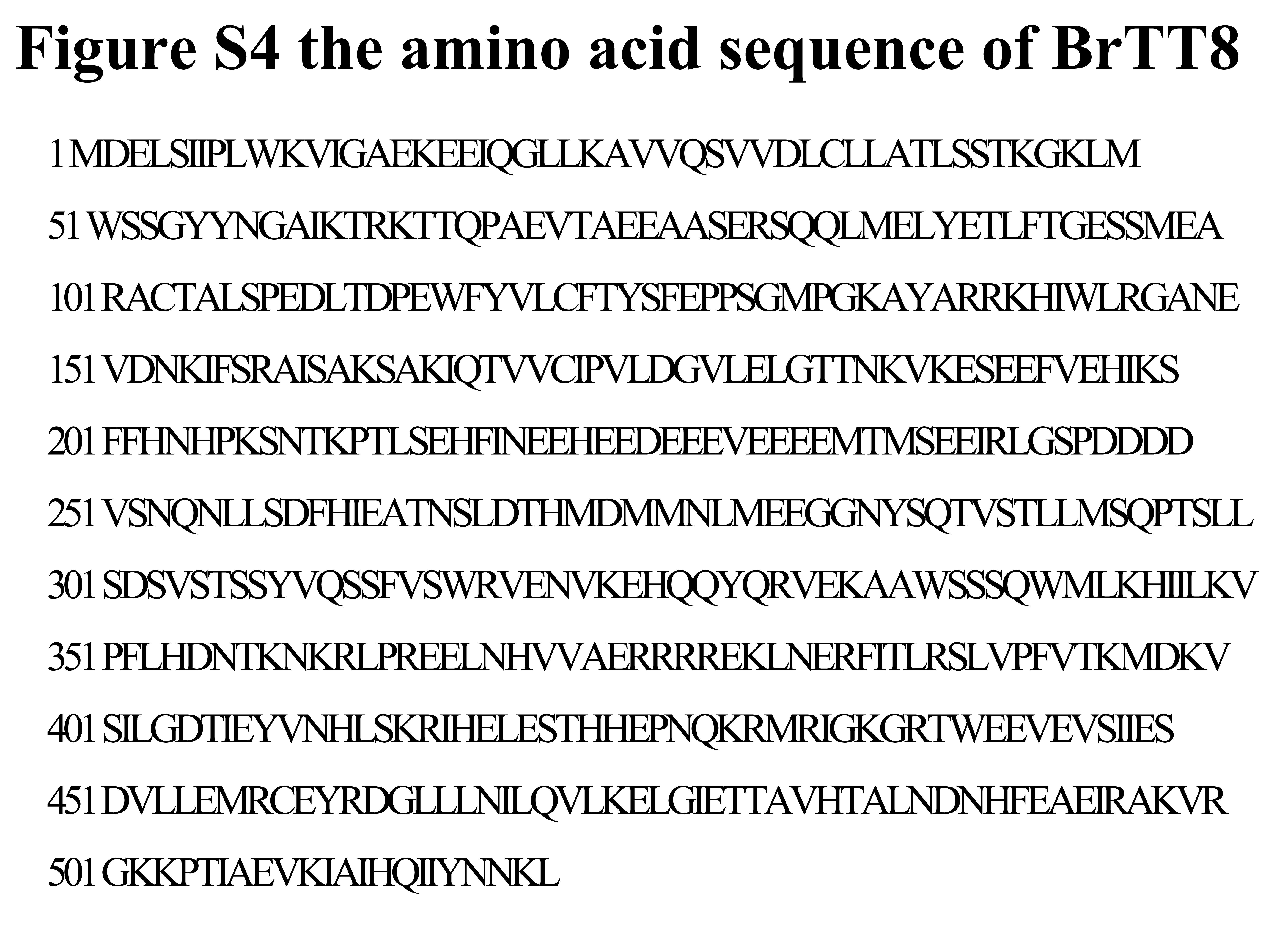

Supplement: Figure S4 — The amino acid sequence of BrTT8. (TIF) [file pone.0044145.s004.tif]
