# Supplementary material for: A Large Insertion in bHLH Transcription Factor BrTT8 Resulting in Yellow Seed Coat in Brassica rapa
Source: PLoS One. 2012 Sep 11;7(9):e44145. doi: 10.1371/journal.pone.0044145 (PMC3439492; doi:10.1371/journal.pone.0044145)
Supplement: Table S1 — The SSR markers were developed in the study. (DOC) [file pone.0044145.s005.doc]

Table S1 The SSR markers were developed in the study
