# Supplementary material for: A Large Insertion in bHLH Transcription Factor BrTT8 Resulting in Yellow Seed Coat in Brassica rapa
Source: PLoS One. 2012 Sep 11;7(9):e44145. doi: 10.1371/journal.pone.0044145 (PMC3439492; doi:10.1371/journal.pone.0044145)
Supplement: Table S2 — The primers were used in the study. (DOC) [file pone.0044145.s006.doc]

| **Table S2 The primers were used in the study** | |
| --- | --- |
| **primer name** | **primer sequence** |
| Tu-L | 5’-3’ GAACTGACATAATCCTGAAGCAC |
| Tu-R | 5’-3’ CCATTACACACGTAAGCCTTCTT |
| TL1 | 5’-3’TAAGGCGGTGGTGCAATCTG |
| TR1 | 5’-3’ CTTGTTCGTTGTGCCTAGTTCC |
| TL2 | 5’-3’TGGTTTTATGTGCTGTGTTTCACT |
| YCR1 | 5’-3’CGACGGAAGTTACGGCTGAAGAG |
