# Supplementary material for: A Large Insertion in bHLH Transcription Factor BrTT8 Resulting in Yellow Seed Coat in Brassica rapa
Source: PLoS One. 2012 Sep 11;7(9):e44145. doi: 10.1371/journal.pone.0044145 (PMC3439492; doi:10.1371/journal.pone.0044145)
Supplement: Table S3 — The primers were used for Q-PCR. (DOC) [file pone.0044145.s007.doc]

| **Table S3 The primers were used for Q-PCR** | |
| --- | --- |
| **primer name** | **primer sequence** |
| RTBANL | 5'-3’TTAACTGGGCATACCCAATCTC |
| RTBANR | 5'-3’TGCATTTCTTTCCGGGTAATC |
| RTTT3L | 5'-3’CAGGATGGATGTATTTCATGTCG |
| RTTT3R | 5'-3’TGTGCCTCGTTACGAGTGATAG |
| RTTT6F | 5'-3’CGAGAAAGAGGCACTCACCAATG |
| RTTT6R | 5'-3’TGAACCTCCCGTTGCTCAGATA |
| RTTT7F | 5'-3’GTGGTTGCCGCCTCTAAATC |
| RTTT7R | 5'-3’CTAGCTCGCGCATGAGTGTTC |
| RTLDOXF | 5'-3’ACGCGAGTGGACAGCTTGAGT |
| RTLDOXR | 5'-3’GCGATAGAGAGAGCCTTGAAGAC |
| actinF | 5'-3’CTATCCTCCGTCTCGATCTCGC |
| actinR | 5'-3’CTTAGCCGTCTCCAGCTCTTGC |
